# Mental Health in Urban Environments: Uncovering the Black Box of Person-Place Interactions Requires Interdisciplinary Approaches

**DOI:** 10.2196/41345

**Published:** 2023-05-11

**Authors:** Martina Kanning, Li Yi, Chih-Hsiang Yang, Christina Niermann, Stefan Fina

**Affiliations:** 1 Department of Sport Science University of Konstanz Konstanz Germany; 2 Department of Nutrition Harvard TH Chan School of Public Health Boston, MA United States; 3 Department of Exercise Science and TecHealth Center, University of South Carolina Columbia, SC United States; 4 Institute of Interdisciplinary Exercise Science and Sports Medicine Medical School Hamburg Hamburg Germany; 5 Faculty of Architecture and Civil Engineering University of Applied Sciences Augsburg Augsburg Germany

**Keywords:** physical activity, urban health, ambulatory assessment, environment, mental health, real-time data, within-subject association

## Abstract

Living in urban environments affects individuals’ mental health through different pathways. For instance, physical activity and social participation are seen as mediators. However, aiming to understand underlying mechanisms, it is necessary to consider that the individual is interacting with its environment. In this regard, this viewpoint discusses how urban health research benefits from integration of socioecological and interdisciplinary perspectives, combined with innovative ambulatory data assessments that enable researchers to integrate different data sources. It is stated that neither focusing on the objective and accurate assessment of the environment (from the perspective of spatial sciences) nor focusing on subjectively measured individual variables (from the public health as well as a psychosocial perspective) alone is suitable to further develop the field. Addressing person-place interactions requires an interdisciplinary view on the level of theory (eg, which variables should be focused on?), assessment methods (eg, combination of time-varying objective and subjective measures), as well as data analysis and interpretation. Firstly, this viewpoint gives an overview on previous findings addressing the relationship of environmental characteristics to physical activity and mental health outcomes. We emphasize the need for approaches that allow us to appropriately assess the real-time interaction between a person and a specific environment and examine within-subject associations. This requires the assessment of environmental features, the spatial-temporal behavior of the individual, and the subjective experiences of the situation together with other individual factors, such as momentary affective states. Therefore, we finally focused on triggered study designs as an innovative ambulatory data assessment approach that allows us to capture real-time data in predefined situations (eg, while walking through a specific urban area).

## Introduction

Globally, 76% of people live in cities, and a growing number of people are expected to move into urban surroundings within the next two decades [1]. Although urban settings are frequently associated with locational advantages (eg, proximity to job and educational opportunities, cultural diversity, as well as service and infrastructure provision), they are also shown by empirical evidence to increase risks for psychological stress and mental disorders among their residents [2]. Even though mental health has complex determinants, theoretical assumptions about urban health and empirical evidence suggest that increasing physical activity levels and social interactions improve mental health (eg, well-being, quality of life, and satisfaction with life) in urban residents [3,4]. Recommendations for creating health-promoting urban environments include the vision of active lifestyles and opportunities for urban residents to participate in social life supported by suitable infrastructure (ie, walkable neighborhoods and people-oriented urban designs). For instance, there is extensive research in the field of walkability in urban areas and its association with health. However, we still have only preliminary insights into people’s reactions to specific environmental features (eg, greenness and noise); we also have limited insight as to for whom and under which conditions such experiences result in more physical activity (eg, active mobility), social interactions, and improved mental health. For these reciprocal associations between the person and its environment, we use the term “person-place interactions,” which former studies were not able to address adequately due to methodological constraints. This shortcoming can be overcome with innovative study designs that allow us to address within-subject relations, and therefore, allow for studying the underlying mechanisms of people’s health behavior and mental health after they have been exposed to different environmental features [5]. In addition, such study designs provide the opportunity to draw from existing and newly emerging data sources and data fusion techniques of different disciplines (eg, spatial science, sport and movement science, and psychology) to get a deeper understanding of person-place interactions.

## Why Do We Need Information About Person-Place Interactions to Create Health-Promoting Urban Environments?

According to socioecological approaches, individuals interact with their physical and natural environment as well as the social neighborhood setting, which affects health-related behaviors such as physical activity [6]. According to Stokols [7], this interaction can be described as “cycles of mutual influences”—the environmental features of a local neighborhood are associated with urban residents’ behavior and health. Reciprocally, individuals live and act within these settings and engage with environmental features in a “more or less” health-enhancing way. For instance, on the one hand, design features of the built environment as well as opportunities to get active in social settings can stimulate physical activity (eg, creating attractive stairs in urban environments). On the other hand, a person who is highly motivated to improve activity levels, experiences and perceives the environment differently than a person who is less motivated, and therefore, acts differently based on these subjective experiences and perceptions (eg, using such stairs not only for stair climbing but also for a workout or to do parkour). Furthermore, according to socioecological approaches, individuals’ behavior is affected by more than just the individual level (eg, motivation, self-efficacy, habits, and personal physiological constitution) and the perceived environment (eg, attractive stairs, perceptions of urban green or blue, and noise) but also by the sociocultural factors, factors arising from the built and the social environment, as well as policy factors [8]. Therefore, learning more about person-place interactions from an interdisciplinary perspective—integrating knowledge from spatial science, psychology, sport science, transport systems, politics, and sociology—is a precondition for creating health-promoting environments. For example, an urban health policy to add cycling lanes to promote physical activity levels may be less effective, if the fit between these environmental features (eg, cycling lanes) and the target groups’ needs, preferences, social-cognitive constructs (eg, attitudes), and sociodemographic backgrounds (eg, age and proportion of bike owners) are not considered. As Stokols [9] emphasized, this fit serves as an important predictor of health and well-being.

## Evidence About the Associations Between the Environment, Physical Activity, and Mental Health

Considering current studies, key findings confirm that environmental features are associated with physical activity and health. An analysis of previous systematic reviews and meta-studies [10] summarizes associations between built environmental features, dietary intake, physical activity, and obesity. More than half of the included reviews focused on physical activity (n=46) and reported consistent evidence about the positive associations between walkability and physical activity (supported by 83% of the reviews), followed by positive associations between access to recreational facilities, shops and services, and parks or trails and physical activity (supported by 63% to 70% of the reviews). Another systematic review of longitudinal studies (N=36) about the effects of the built environment on adults’ physical activity came to similar conclusions: new infrastructures for walking, cycling, and using public transport increase overall physical activity [11]. Further reviews, especially in the past few years, support such results for older adults [12] and children [13].

In terms of mental health impacts, an overview of systematic reviews assessed the association between the built environment and different mental health indicators (eg, well-being, depression, and stress) [14]. The authors included 11 reviews and reported insufficient and heterogeneous evidence for health-enhancing effects of the environment, with a critically low methodological study quality of 80% of the included reviews. Another meta-narrative review synthesizes the impacts of urban green space on different indicators of mental health from 38 intervention studies [15]. The results were discussed in an international World Health Organization expert panel workshop, concluding that urban green space interventions are often multifaceted but can generally be categorized in 4 groups: park-based, greenways and trails, urban greening (eg, street trees), and green built interventions (eg, green roofs). Most studies in that meta-narrative review were designed as natural experiments, and the findings showed strong evidence for park-based as well as greenway and trail interventions to promote health and well-being through increased park use and physical activity [15].

## Limitations of the Empirical Evidence When Analyzing Person-Place Interactions

The aforementioned reviews provide evidence about the relationships between specific environmental features and mental health or physical activity. However, they fall short of increasing our knowledge of the time-varying associations within subjects regarding how urban residents react to specific environmental features and under which conditions such experiences result in more physical activity and improved mental health. One reason for this is that these reviews focused mainly on environmental characteristics, such as accessibility or the amount or quality of greenness, and how these characteristics moderate the relationship between the environment and physical activity or mental health. They do not provide evidence about individuals’ momentary perception, experience, and subsequent behavioral, cognitive, or affective states; nor do they show how these states are related to mental health.

Kwan [16] already criticized in 2009 that spatial research about associations between environmental features and physical activity or health mainly used a “place-based” instead of a “person-based” approach and operationalized environment exposure mostly by focusing on spatial units, such as census tracts, buffer zones, or postal codes. Such a “place-based” design neglects that individuals move around and do not stay in their “home spatial unit” during their daily activities (eg, workplace, school, and leisure activities) [17].

More than 10 years later, Zhang and colleagues [18] stated that there are still only a few studies investigating the association between environment and health from the perspective of the spatial-temporal behavior of the individual. According to the results of their survey with 1003 Chinese adults, there are significant differences between environmental exposures of individuals based on home buffer zones (ie, place-based) compared to time-weighted activity travel buffers (ie, person-based) [18].

A currently published scoping review [19] also stated that person-based approaches still are in their infancy. The review is about methodological approaches to measure the spatial contexts used in socioecological physical activity research, and the included studies have been mostly published within the last 7 years. In sum, person-based spatial methods have been used rarely; only 2% (10/412) of the included studies used activity spaces, and similarly, only 2% (8/412) of the studies buffered multiple points to capture the environment. Almost a third of the studies (118/412) used place-based approaches (eg, with administrative units) as an objective approach.

Furthermore, place-based approaches do not take into consideration that individuals have different lifestyles, psychosocial characteristics, and daily routines and may react differently to influences of similar environmental features. For instance, even persons living within the same building would perceive environmental exposure of their neighborhood differently [20]. That refers to the “uncertain geographic context problem” [21], and it also highlights the importance of interdisciplinary efforts by psychologists, sport scientists, geographers, and computer scientists [7,22]. Nevertheless, empirical evidence of interdisciplinary studies integrating assessment methods of spatial science and urban planning as well as social and health sciences is still lacking [23]. Further, these approaches do not allow detailed analysis; for example, to what extent social interaction could moderate the associations between the environment and mental health or for whom the quality of greenness may be relevant. They also neglect that the environmental exposure is not only directly associated with mental health but also via affecting health-related behaviors, such as physical activity. Thus, mediators or moderators of the associations between the environment and physical activity and health have hardly been examined so far [10]; a framework to explore relationships between place and mental health by combining GPS, Geographic Information System (GIS), and accelerometer data is available in a previous study [24].

## Ambulatory Assessment Approaches Are Suitable to Analyze Person-Place Interactions

To advance our understanding of person-place interactions of urban residents in everyday life, we need more studies that collect intensive longitudinal data, which facilitates the estimation of time-varying associations between environmental features, individuals’ behavior, as well as their momentary experiences. Ambulatory assessments are suitable approaches for addressing such within-subject relations because they allow us to monitor physical activity (eg, via accelerometry), physiological function (eg, heart rate or electrodermal activity), and environmental parameters (eg, via geolocation tracking) in real time.

In 2018, Chaix [23] published an overview of different wearable sensors and devices to capture the environment (eg, air pollution and the number of mobile phones nearby), the behavior (eg, physical activity and GPS receivers), and individuals’ physiology (eg, heart rate and electrodermal activity). He recommended integrating different sensors to generate knowledge of healthy places and situations. Such an approach allows us to assess the duration, sequences, and accumulation of different environmental exposures; it also provides rich research possibilities to assess in situ changes in mental health according to different environments or environmental features [25].

A current example of combining different sensors is the study by Marquet and colleagues [26], which combined accelerometers to assess physical activity and GPS data that was linked to spatial data sets on walkability and greenness. They found that persons with high walkability and greenness in their activity spaces had higher levels of moderate-to-vigorous physical activity. In addition, a recent study [27] combined sensors that measure black carbon concentration with a sensor that assessed galvanic skin response (as a proxy measure of stress) and a GPS device and found that increases in black carbon are related to higher skin responses (indicating higher stress levels) during active travel. Green space and a good active travel infrastructure are associated with lower skin responses while walking or cycling.

To deepen our understanding of how different individuals react to specific environmental features, it is crucial to assess the above-mentioned environmental parameters and physiological functions using sensors in an objective way; however, it is also relevant to assess subjective experiences, preferably at that moment when an association between a person and a place is assumed. It is possible to schedule e-diaries throughout the day (eg, ecological momentary assessment) to assess different psychosocial constructs (eg, momentary affective states, momentary experiences of social interactions, and momentary motivation) via self-reported measures [28,29].

Ambulatory assessment approaches have already been applied in spatial research. Perchoux et al [30,31] introduce activity spaces as an individualized measure for environmental exposure. It considers individuals’ daily mobility patterns, including major spatial-temporal cluster movements between home and different daily locations, and characterizes its temporal structure (ie, frequency, regularity, and duration). Further, to match person- and place-based data, some research groups combined the assessment of activity spaces with multiple self-reports per day via e-diaries (eg, feelings, emotions, and evaluations of their environment) resulting in geographically explicit ecological momentary assessments (GEMAs) [5,20]. GEMA studies implement innovative study designs that use mobile geographic location technologies to capture participants’ activity space, which can then be used to assess the dynamic environmental exposure via GIS. e-Diaries allow us to capture subjective experiences in situ, and these data can be linked to participants’ current position in time and space. However, GEMA struggles with the disadvantages of “time-based” assessments because prompts to answer the web-based questionnaires were usually triggered at random time intervals. With such a sampling scheme, self-reports during rare events (eg, being physically active in the neighborhood) are likely to be missed and could therefore hardly be used for analyzing time-varying associations. For instance, a GEMA study [32] assessed how urban green space is associated with stress in adolescents living in urban surroundings. Outdoor behaviors were assessed via GPS-enabled mobile phones. To capture momentary experiences of stress, participants received randomly 3-6 text messages throughout the day, including a link to a web-based questionnaire. However, 72% of the web-based questionnaires have been filled in at home and not during outdoor behaviors. To ask participants to report retrospectively about their feelings, experiences, and thoughts in response to specific situations is likely to increase recall bias, for instance [29]. Furthermore, spatial accuracy of GEMA is an issue and should be taken into account when analyzing and interpreting the data in urban settings when the GPS device signals are likely to be interrupted, such as in streets with dense tree covers [33].

## Methodological Improvements and Future Directions of Research

Answers to research questions, such as the following, are crucial to inform initiatives aiming to create health-promoting urban environments: “How does mental health and physical activity vary due to the momentary exposure to specific physical (eg, streetscape greenery and noise) or social environmental features (eg, crime or places enabling social interactions)?” and “How are these time-varying associations moderated by personal factors, which could be time-invariant, such as lifestyle, attitudes, socioeconomic status, obesity, and gender, as well as dynamic such as momentary feelings, experiences, motivations, and thoughts?”

To address these questions, we need data of within-subject relations during predefined situations in everyday life in which a contextual effect is assumed (eg, being physically active within a neighborhood). The above-mentioned GEMA approach is an appropriate design to provide first answers. However, to capture data during the “right” situations, it could be extended by (1) assessing physical activity directly via accelerometers and (2) using triggered assessments. Triggered assessments allow capturing data in predefined situations and have already been applied in other fields, such as examining time-sensitive associations between physical activity and affective states [34] or the time-sensitive assessment of contextual factors during episodes of prolonged sedentary bouts [35]. A recent example of a triggered e-diary regarding outdoor activities was presented in a study protocol with older residents of Paris [36]. The study combined GEMA with a GPS receiver and used this novel methodology to initiate e-diaries when participants were outdoors. Another study used momentary physical activity levels (assessed via accelerometry) and locations by mobile phone positioning services (eg, GPS and transmission tower) to identify outdoor activities. A trigger algorithm was used to start an e-diary whenever movement acceleration exceeds a certain threshold and participant’s locations were identified as outside the home [37]. The study included 46 middle-aged adults and showed that momentary affective states varied significantly due to different social (intensity of social interaction) and physical (amount of greenness) environments. The accuracy of the walking trigger has been examined in a previous study [38].

Furthermore, activity data could be integrated into a GIS to combine information of the physical environment of the activity spaces with movement data. Advanced GISs work with time-enabled spatial analysis functions to track movements with so-called “event-based feature classes.” The challenge is to provide data on the actual exposure for the time of measurement. In this context, live sensor networks are preferable over archived data but only starting to become available, for example, in Smart City Sensor Observation Networks [39,40]. Through combining different data (eg, subjective experiences via self-reports and physical activity levels and physiological functions via specific sensors) in outdoor situations with exposures to different environmental features, we would be able to investigate the associations between specific uses of the environment (eg, walking, social interactions, and doing sports) and momentary experiences (eg, reduced stress and better feelings) and how the characteristics of the environment, the living conditions, or psychological factors moderate these associations.

Despite its promising future perspectives, these kinds of person-based spatial approaches lead to several methodological challenges concerning data processing, the linking of spatial and contextual exposures to individuals, special analytical and statistical methods, and ethical aspects of participants’ privacy and security [41,42]. The solution to these challenges calls for the assembly of an interdisciplinary research team, which itself might also be challenging. However, this approach would enable us to take a broader perspective on this phenomenon and get closer to draw a “bigger picture” of person-place interactions during the everyday life of urban residents.

## Conclusions

Urbanization with its advantages and disadvantages concerning health is on the rise. This viewpoint paper highlights the importance of gaining knowledge regarding the effect of urban environments on people’s mental health by considering socioecological and interdisciplinary perspectives in combination with triggered ambulatory data assessments. It is crucial to assess time-varying associations to investigate person-place interactions between the individual and physical, social, and contextual features. Progress in technology and methodological advances enables researchers to study in more detail how people react to specific environmental features and which situational or personal factors may moderate these associations. Lastly, combining data and knowledge of different disciplines would deepen our understanding about the person-place interactions, which is crucial to create health-promoting urban environments.

## Abbreviations

GEMA: geographically explicit ecological momentary assessment
GIS: geographic information system
